# Challenges and advances in prenatal diagnosis and management of beta thalassemia in Pakistan

**DOI:** 10.1097/BS9.0000000000000244

**Published:** 2025-07-02

**Authors:** Muhammad Suleman Ur Rehman, Sadiq Murtaza Sheriff, Muhammad Mahad Riaz, Muhammad Areeb Ul Haq

**Affiliations:** a Department of Medicine, Dow Medical College, Karachi, Pakistan; b Department of Medicine, Allama Iqbal Medical College, Lahore, Pakistan

## To The Editor:

Beta thalassemia is a genetically transmitted blood disorder with devastating outcomes for developing countries like Pakistan, largely due to inadequate health policies and infrastructure. Pakistan has a population of approximately 225 million and a β-thalassemia major (β-TM) carrier frequency of over 5%, resulting in more than 9 million carriers nationwide.^1^

Thalassemia is characterized by mutations in the hemoglobin chain, leading to impaired oxygen transport. For accurate molecular diagnosis, essential tests include complete blood count (CBC), hemoglobin electrophoresis, chorionic villus sampling (CVS), polymerase chain reaction (PCR), and amniocentesis.^2^ Treatment typically involves iron chelation and bone marrow transplantation. While these interventions are lifesaving, prevention strategies like population screening, genetic counseling, and prenatal diagnosis are more sustainable solutions.^1^

There are several challenges in the prenatal diagnosis of beta thalassemia in Pakistan. Techniques like CVS and amniocentesis are invasive, time-consuming, and associated with a risk of miscarriage.^2,3^ An alternative approach involves using maternal plasma to isolate cell-free fetal DNA (cff-DNA), which can be rapidly and accurately analyzed using digital PCR (d-PCR), enhancing diagnostic sensitivity and accuracy.^2,4^ However, the extremely low quantity of cff-DNA in maternal plasma limits its practical use in prenatal diagnosis.^2^

Relative Haplotype Dosage (RHDO) is another technique that detects parental mutations by analyzing single nucleotide polymorphisms (SNPs). While RHDO has proven effective in European countries,^5^ its application in Pakistan could be promising—especially if consanguineous marriages are discouraged and next-generation sequencing (NGS)–based diagnostics are adopted. Yet, high costs, need for specialized equipment, and technical expertise present significant barriers.^2^ For couples undergoing in vitro fertilization (IVF), preimplantation genetic diagnosis can help prevent beta thalassemia by identifying and screening for chromosomal abnormalities, thereby reducing the need for abortion—a practice considered ethically and socially contentious in Pakistan.^3^

An estimated 5000 to 9000 children are born with beta thalassemia annually in the country.^1^ These individuals require lifelong blood transfusions, which can lead to iron overload, elevated liver enzymes, gastrointestinal disorders, and arthralgia. This makes treatment with iron-chelating drugs such as deferasirox (DFX), deferiprone (DFP), and deferoxamine (DFO) necessary. Organizations such as the Patient Welfare Association (PWA) and the Al Zaib Foundation are actively working to address this burden. Bone marrow transplantation remains the only curative treatment, and Pakistan currently has 12 operational centers offering this procedure, with an overall survival rate of about 80%.^6^ Non-governmental organizations (NGOs) like the Afzal Memorial Thalassemia Foundation (AMTF) and the Punjab Thalassemia and Other Genetic Disorders Prevention and Research Institute provide essential financial support to manage the associated expenses.

To improve outcomes, research into amplifying cff-DNA via PCR could expand the use of noninvasive prenatal testing.^2^ Moreover, genetic counseling can help mitigate the adverse effects of consanguineous marriages—an underemphasized issue despite genetics being a mainstream subject in Pakistan’s biological sciences education. Effective collaboration between the government, NGOs, and the public health sector is crucial for timely decision-making and improved patient outcomes.

Recent developments in gene therapy have shown promising results for beta thalassemia. The Food and Drug Administration (FDA)-approved gene therapy betibeglogene autotemcel (Zynteglo) introduces a functional β-globin gene into the patient’s hematopoietic stem cells, enabling endogenous production of normal hemoglobin and significantly reducing or eliminating the need for transfusions.^7^ Additionally, CRISPR-Cas9–based approaches like exagamglogene autotemcel (Casgevy)^8^ target the *BCL11A* gene to reactivate fetal hemoglobin (HbF) synthesis, offering a potential one-time curative solution. These therapies mark a paradigm shift, though their high cost and the specialized infrastructure required for their application are significant challenges for low-resource countries like Pakistan.

Raising public awareness through social media campaigns and educational programs in colleges and universities about the importance of thalassemia screening is vital. With focused attention and coordinated efforts, Pakistan can make significant strides in disease prevention and management. Every child born free of this condition represents a triumph of science, policy, and public resolve.

## AUTHOR CONTRIBUTIONS

M.S.U.R. participated in the writing of the paper (first paragraph), conducted a literature search, and contributed to referencing. M.M.R. participated in the writing of the paper (second and third paragraphs) and contributed to the final editing of the manuscript. S.M.S. conceived the topic idea, participated in the research design, and wrote the fourth paragraph of the manuscript. M.A.U.H. participated in the review and proofreading of the manuscript; contributed to editing, referencing, and submission; and proposed the title.
